# Different Mutation Tolerance of Lentiviral (HIV-1) and Deltaretroviral (BLV and HTLV) Protease Precursors

**DOI:** 10.3390/v14091888

**Published:** 2022-08-26

**Authors:** János András Mótyán, Norbert Kassay, Krisztina Matúz, József Tőzsér

**Affiliations:** 1 Department of Biochemistry and Molecular Biology, Faculty of Medicine, University of Debrecen, 4032 Debrecen, Hungary; 2 Doctoral School of Molecular Cell and Immune Biology, University of Debrecen, 4032 Debrecen, Hungary

**Keywords:** human T-lymphotropic virus, bovine leukemia virus, human immunodeficiency virus, HTLV, BLV, HIV-1, protease, autoproteolysis, retrovirus, retroviral protease

## Abstract

The bovine leukemia virus (BLV) and the human T-lymphothropic viruses (HTLVs) are members of the deltaretrovirus genus of *Retroviridae* family. An essential event of the retroviral life cycle is the processing of the polyproteins by the viral protease (PR); consequently, these enzymes became important therapeutic targets of the anti-retroviral drugs. As compared to human immunodeficiency viruses (HIVs), the deltaretroviruses have a different replication strategy, as they replicate predominantly in the DNA form, by forcing the infected cell to divide, unlike HIV-1, which replicates mainly by producing a vast number of progeny virions and by reinfection. Due to bypassing the error-prone reverse transcription step of replication, the PRs of deltaretroviruses did not undergo such extensive evolution as HIV PRs and remained more highly conserved. In this work, we studied the abilities of wild-type and modified BLV, HTLV (type 1, 2 and 3), and HIV-1 PRs (fused to an N-terminal MBP tag) for self-processing. We designed a cleavage site mutant MBP-fused BLV PR precursor as well, this recombinant enzyme was unable for self-proteolysis, the MBP fusion tag decreased its catalytic efficiency but showed an unusually low K_i_ for the IB-268 protease inhibitor. Our results show that the HTLV and BLV deltaretrovirus PRs exhibit lower mutation tolerance as compared to HIV-1 PR, and are less likely to retain their activity upon point mutations at various positions, indicating a higher flexibility of HIV-1 PR in tolerating mutations under selective pressure.

## 1. Introduction

The retroviruses belong to the family of *Retroviridae* of Group IV viruses. Most retroviruses are classified into the *Orthoretrovirinae* subfamily (α-, β-, γ-, δ-, and ε-retroviruses, and lentiviruses), while the spumaviruses belong to the *Spumaretrovirinae* subfamily [1]. The human immunodeficiency virus (HIVs) and the human T-cell leukemia virus (HTLVs) are the most relevant human pathogenic retroviruses, and multiple subtypes have been identified to date, such as HIV-1/2 and HTLV-1/2/3/4. The HIVs (HIV-1 and HIV-2) belong to the *Lentivirus* genus and are the causative agents of acquired immune deficiency syndrome (AIDS). The HTLV viruses belong to the *Deltaretrovirus* genus. The conventional classification of the primate T-lymphotropic viruses (PTLVs) is based on the host of origin, viruses infecting humans (HTLVs) and non-human primates (simian T-lymphotropic viruses, STLVs) can be differentiated. The HTLV infection is known to be correlated to the development of adult T-cell leukemia/lymphoma and tropical plastic paraparesis [1]. The bovine leukemia virus (BLV) is also a member of the *Deltaretrovirus* genus; it is closely related to HTLV-1/2 and it is the causative agent of enzootic bovine leucosis, a disease characterized by the occurrence of the clonal lymphoid tumor of B-cell origin [2]. Humans are also exposed to the BLV infection; the human cells may be susceptible to BLV due to the transmission of the virus via consumption of infected foodstuffs [3,4], but the correlation of BLV with the development of diseases such as breast cancer is still controversial [5]. BLV is considered to be a valuable model system for understanding the HTLVs.

Based on the 2021 year report of UNAIDS the number of people living with HIV is 37.7 million [6], while according to the 2015th year report of European Centre for Disease Prevention and Control [7] the number of HTLV-1-infected people worldwide is 5–10 million, but the estimated number of infected people may be higher and even 20 million [8,9,10]. The data on prevalence are thought to be underestimated because the data were obtained from studies of the most endemic regions and have been performed in different times, making the estimation of the recent numbers of the global epidemic unreliable [11]. The data about the worldwide prevalence of all HTLV viruses are limited and available mainly for HTLV-1 [12], the number of HTLV-2-infected people is lower and is estimated to be between 0.7 and 0.9 million globally [13]. The BLV is widespread in all continents, with the highest prevalence in the United States and the lowest in European countries [14].

Lentiviruses, such as HIV and deltaretroviruses (including BLV and HTLVs), share the main steps of their life cycles. Both lenti- and deltaretroviruses are enveloped viruses which have a single-stranded (+)RNA genome. Their genomes contain three main coding regions: *gag*, *pol*, and *env*. The *gag* gene codes for the matrix (MA), capsid (CA), and nucleocapsid (NC) structural proteins. The *pol* encodes the viral enzymes, the reverse transcriptase (RT), the integrase (IN), and the protease (PR). The surface glycoprotein (SU) and transmembrane (TM) proteins are part of the envelope precursor polyprotein and are coded by the *env* gene [15]. Both lentiviruses and deltaretroviruses are complex retroviruses, and they encode not only structural proteins and replicative enzymes but regulatory and accessory proteins as well. The first step of the retroviral replication cycle is the receptor-mediated attachment of the virion to the target cell. A unique feature of the *Lentivirus* genus members as compared to other retroviruses is that they can infect not only dividing but also non-dividing cells [16]. The fusion of the viral envelope and the target cell’s membrane is followed by the entry, then the retroviral DNA is synthesized from the genomic RNA. This is carried out by the RT; the reverse transcription is the most error-prone step of the HIV life cycle [17]. The main difference between the life cycles of retroviruses and other viruses is that the retroviral life cycles include the integration of the viral DNA into the genome of the infected cell, the irreversible insertion of the viral DNA is catalyzed by the IN enzyme, making the permanent production of the replication-competent virions from the proviruses possible [18]. The integrated proviral DNA is used to transcribe the viral RNA molecules, which can be used as templates to translate the viral polyproteins. During assembly, the immature virions are formed from the genomic RNA and the viral proteins. Then, the immature virions undergo maturation via the proteolytic processing of the polyproteins into functional units by the PR [14,19,20].

The HIVs and HTLVs show characteristic differences in their replication strategies. The HIVs produce a large number of progeny virions and replicate by reinfection. Due to the highly error-prone nature of reverse transcription, mutations are frequently introduced into the viral genome, i.e., the large genetic diversity—which is characteristic for HIV—is caused by the extensive reverse transcription [17]. In contrast to HIV, the HTLVs exhibit much lower genetic variation. The replication of HTLV-1 is strongly cell-associated, the virus is spread between the T-cells via cell–cell contacts, also referred to as virological synapses [21]. The HTLVs replicate predominantly in the DNA form, forcing the infected cells to divide; therefore, the high viral load is achieved by relatively fewer reverse transcription steps. HTLVs have much more conserved genome due to bypassing the reverse transcription step of replication; consequently, they did not undergo such extensive evolution as HIVs [22,23,24,25]. The preferred replication strategies are considered to be determined in part by the accessory and regulatory proteins (Tax and Tat, and Rex and Rev in HTLV-1 and HIV-1, respectively) [22].

The sequence variations of the HIV-1 PR and its tolerance to mutations is well characterized by multiple studies on drug-induced resistance mutations and the circulating natural sequence variants [26,27,28,29,30,31]. Numerous mutations of deltaretroviruses have also been studied [32], but the information about the sequence variations and resistance mutations of HTLV and BLV PRs are more limited as compared to HIV PRs. Specificity studies using a series of oligopeptide substrates revealed that the HTLV PRs have a more rigid specificity as compared to BLV and HIV-1 PRs, and the effects of mutations on the self-processing ability have already been investigated in the case of BLV [33], HTLV-1 [34], and HTLV-2 and HTLV-3 PRs [35]. In these studies, the auto-proteolysis was investigated using MBP-fused enzymes that were linked to the N-terminal fusion tag by a short linker representing a natural autoproteolytic cleavage site sequence. 

This simple experimental system is useful to investigate the effects of protease and/or cleavage site mutations by determining the abilities of the wild-type and mutant enzymes for self-processing. In this work, we studied the mutation tolerance of the proteases of such retroviruses which have a different replication strategy and genetic stability, especially that of a lentivirus (HIV-1) and multiple deltaretroviruses (HTLV-1, HTLV-2, HTLV-3 and BLV).

## 2. Materials and Methods

All materials were purchased from Sigma-Aldrich (St. Louis, MI, USA), unless otherwise indicated.

### 2.1. Expression Constructs and Mutagenesis

The expression plasmids used in this study are shown in Table 1. The expression constructs encoded the proteases fused to an N-terminal maltose-binding protein (MBP) fusion tag. The MBP and the protease domains were connected by an 8 residue-long linker representing the P8-P1 residues of the natural N-terminal autoproteolytic cleavage site sequence of the corresponding PR. The expression plasmid coding for the wild-type HIV-1 PR was in-house stock [36], this plasmid was modified by insertion of an N-terminal flanking sequence by overlap extension PCR as it is described below. The plasmid for the expression of the HIV-1 PR containing five stabilizing mutations (HIV-1 PR5) was obtained from a gene synthesis service (Genscript Biotech, Piscataway, NJ, USA). The expression construct coding for the wild-type HTLV-1 PR was in-house stock [34]. The coding sequence of the HTLV-1 PR containing three stabilizing mutations of the protease (HTLV-1 PR3, it was also referred as C2A-HTLV-1 PR) [37] was cloned into pMalc5x vector. The expression constructs coding for the MBP-fused BLV [38], HTLV-2 and HTLV-3 PRs [35] were in-house stocks.

Mutations of the MBP-fused PRs were generated by overlap extension PCR. The mutagenesis and overlap primers are listed in Table S1. The overlap PCR consists of two PCR steps. In the first step, two primers are used to generate DNA fragments that contain the desired mutation and have overlapping ends. Then, the spliced PCR products are used as template in a second PCR reaction to produce the hybrid duplexes. The PCR products and the plasmids were digested by restriction endonucleases (Table 1), followed by ligation using T4 Ligase (Thermo Fisher Scientific, Waltham, MA, USA). The success of cloning was verified by sequencing (Eurofins Genomics sequencing service), using standard MalE primer (TCAGACTGTCGATGAAGC).

The pMAL-c2 BLV PR clone—encoding the BLV PR [38]—was used as a template for the introduction of P2 and P1 substitutions of the N-terminal flanking sequence. Mutagenesis was performed by using a QuickChange mutagenesis kit (Stratagene, Agilent Technologies, Santa Clara, CA, USA) with appropriate oligonucleotide pairs (5′-GTGAGGCCGAATTAGAAGGCGGACTTTCTATTCCTCTTGGC-3′ and 5′-GCCAGAGGAATAGAAAGTCCGCCTTCTAATTCGGCCTCAC-3′). The success of mutagenesis was verified by DNA sequencing performed with an ABI Prism Dye Terminator cycle sequencing kit and model 373 A sequencer (Applied Biosystems, Foster City, CA, USA). The amplified DNA fragments were cloned into pMal-c2 vector (New England Biolabs, Ipswich, MA, USA), after the MBP gene.

### 2.2. Transformation and Expression of MBP-Fused Proteases

The expression plasmids coding for the MBP-fused PRs were transformed into BL21(DE3) *E. coli* cells (New England Biolabs, Ipswich, MA, USA) using heat-shock at 42 °C for 90 s. The transformed cells were cultured in Luria–Bertani (LB) medium supplemented with ampicillin and were grown at 37 °C while shaking until the optical density measured at 600 nm wavelength became 0.6–0.8. The protein expression was induced by the addition of isopropyl β-D-1-thiogalactopyranoside (IPTG, in 1 mM final concentration), followed by incubation at 37 °C for 3 h while continuously shaking.

### 2.3. Expression and Purification of the Cleavage Site Mutant BLV PR

The plasmid coding for the BLV PR2 was transformed into DH5α *E. coli* cells. The cells were grown at 37 °C in Luria–Bertani medium supplemented with 100 mg/mL ampicillin. The protein expression was induced by the addition of 1 mM IPTG, followed by incubation of the cells for 2 h. 

For large-scale purification, the cells were harvested by centrifugation and suspended in lysis buffer (50 mM Tris-HCl, 1 mM ethylenediaminetetraacetic acid (EDTA) and 100 mM NaCl, 1 mM phenylmethylsulfonyl fluoride (PMSF), pH 8.0). Then, the cells were lysed sonication, as it was described previously [38]. The inclusion bodies were washed with lysis buffer containing 0.5% Triton X-100, and the pellet was dissolved in 100 mM Tris-HCl buffer (pH 8.0) containing 6 M guanidine-HCl, on ice. The samples were dialyzed stepwise against 100 mM Tris-HCl (pH 8.0) containing 2 M guanidine-HCl, then against buffer 20 mM Tris-HCl (pH 7.2) containing 100 mM NaCl, 1 mM EDTA, 5% glycerol, and 0.1% Triton X-100. Finally, the solution was clarified by centrifugation and filtered through a 0.22 μm membrane. 

After solubilization, the MBP-fused BLV PR2 was purified by amylose affinity chromatography column (New England Biolabs, Ipswich, MA, USA) using binding (20 mM Tris-HCl, 100 mM NaCl, 1 mM EDTA, pH 7.2) and elution (20 mM Tris-HCl, 1 M NaCl, 1 mM EDTA, pH 7.2) buffers. The eluted fractions were used for kinetic measurements. 

### 2.4. Western Blot

After protein expression, the cells were collected by centrifugation (5000× *g*, 5 min, 4 °C), and lysed by sonication (Branson Sonifier 450, Emerson Electric, Sterling Heights, MI, USA) in lysis buffer (50 mM Tris-HCl, 1 mM dithiothreitol (DTT), 1 mM EDTA, pH 8.2). The cell lysates were supplemented with 2× sample loading buffer (supplemented with SDS and DTT) and incubated at 95 °C for 10 min. The samples were loaded onto 12% polyacrylamide gel and separated at 100 V voltage. After SDS-PAGE, proteins were transferred onto a nitrocellulose membrane (100 V, 1 h) and the proteins were detected by Western blot. The membrane was incubated in 5% dry milk in Tris-buffered saline (TBS, 20 mM Tris-HCl, 0.5 M sodium chloride, pH 7.5) for 1 h at room temperature. The primary anti-MBP monoclonal antibody (E8030S, 1:40,000) (New England Biolabs, Ipswich, MA, USA) was diluted in Tris-buffered saline complemented with 0.05 *v*/*v*% Tween20 (TTBS, pH = 7.5) containing 5% dry milk and incubated with the membrane overnight at 4°C while continuously shaking. The membranes were washed three times with TTBS for 15 min and were then incubated with anti-rabbit horseradish peroxidase (HRP)-conjugate (BioRad, Hercules, CA, USA) (170–6515, 1:10,000) secondary antibody for 1 h at room temperature. The membranes were washed again with TTBS in repeated steps and then the proteins were detected on the membranes using SuperSignal West Pico PLUS chemiluminescent substrate (Thermo Fisher Scientific, Waltham, MA, USA). The membranes were documented using Azure 600 Imaging System (Azure Biosystems, Dublin, CA, USA). The Western blot analyses were performed in at least three replicates in the case of each enzyme.

In the case of BLV PR2, the Western blot was performed by using an anti-BLV antibody (prepared from serum of rabbit immunized with chemically synthesized BLV PR [38]), and a peroxidase-conjugated anti-rabbit secondary antibody, using an ECL detection kit (Pierce; Thermo Fisher Scientific, Waltham, MA, USA). 

### 2.5. Enzyme Assay Using Oligopeptide Substrate

An oligopeptide substrate representing the HTLV-1 CA/NC cleavage-site (KTKVL↓VVQPK) was applied for the HPLC-based activity assays based on the protocol described previously [33]. The protease assay was initiated by the mixing 5 μL (6 nM to 330 nM) purified BLV PR2 with 10 μL 2× incubation buffer (0.5 M potassium phosphate containing 10% glycerol, 2 mM EDTA, 10 mM dithiothreitol (DTT), 4 M NaCl, pH 5.6) and 5 μL 0.44 mM substrate. The reaction mixture was incubated at 37 °C for 1 h and the reaction was stopped by the addition of 180 μL 1% trifluoroacetic acid (TFA), followed by injection onto a Nova-Pak C_18_ reversed-phase chromatography column (3.9 × 150 mm Waters Associates Inc.) using an automatic injector. The substrate and the cleavage products were separated by using an increasing water-acetonitrile gradient (0-100%) in the presence of 0.05% TFA. Kinetic parameters were determined by fitting the data obtained at <20% substrate hydrolysis to the Michaelis–Menten equation by using the SigmaPlot Program (SigmaPlot 2001).

For active site titration and inhibition assay, we applied IB-268, a reduced peptide analogue of an HTLV-1 cleavage site (KTKVL-r-VVQPK, where r presents a reduced peptide bond). The IB-268 inhibitor was in-house stock [34], it has been synthesized previously by Dr. Ivo Blaha (Ferring Leciva, Prague, Czech Republic). The concentration of the active enzyme was determined by active-site titration of BLV PR2 using an HPLC-based method described previously for BLV and HTLV-1 PRs [33,39]. In this assay, the volume of substrate KTKVL↓VVQPK was reduced to 4.8 μL (0.25 mM final concentration) and the reaction mixture contained 0.2 μL of IB-268 inhibitor (dissolved in dimethyl sulfoxide, DMSO), as well. The folding efficiency was calculated based on the ratio of the active enzyme (determined by active site titration) and the total protein amount (determined by Bradford assay, Bio-Rad) using the following equation: folding efficiency (%) = (concentration of the active enzyme/concentration of total protein) × 100. At 100% folding efficiency, the protease monomers were considered to be properly folded and form functional homodimers, i.e., all the enzymes that are present in the solution are proteolytically active and the amount of the inactive enzyme (non- or misfolded proteins) is negligible.

## 3. Results and Discussion

### 3.1. Mutation Design

To study how protease mutations affect the autoproteolytic capability of the MBP-fused protease precursors, single point mutations were introduced into N-terminally extended native protease sequences as well as into those containing previously described stabilizing mutations. The rationale behind the mutagenesis of HIV-1 PR was to introduce such residues which correspond to drug-resistance mutations or natural sequence variations. The mutations were selected from the data complied previously [31,40,41,42]. We have selected mainly major resistance mutations of the active site (corresponding to D30N, M46I, I47A, L76V, and I84V mutations of HIV-1 PR), some of the studied resistance mutations are considered to be accessory (L10I, G73S, and V77I). The mutations were not limited to the active site, we modified additional positions which are part of the substrate groove (S-groove) at the enzyme surface (G73S and N88S) [43,44] or do not constitute a part of any substrate binding sites (L10I, V77I, and L90M) [45].

**Figure 1:**
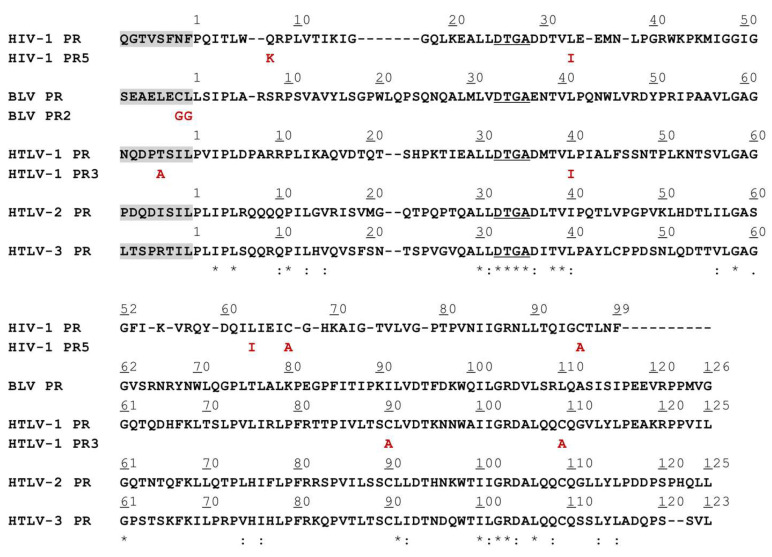
Sequence alignment of HIV-1, BLV, and HTLV proteases. The aligned sequences of HIV-1, BLV, and HTLV proteases are shown. The grey background highlights the N-terminal flanking sequences between the MBP and the protease, these linkers represent the P8-P1 residues of the N-terminal autoproteolytic cleavage site sequences of the corresponding proteases. The residues of the consensus catalytic motif are underlined, the residues that are modified in the fusion proteins are shown by red. Asterisks indicate identical residues.

**Figure 2:**
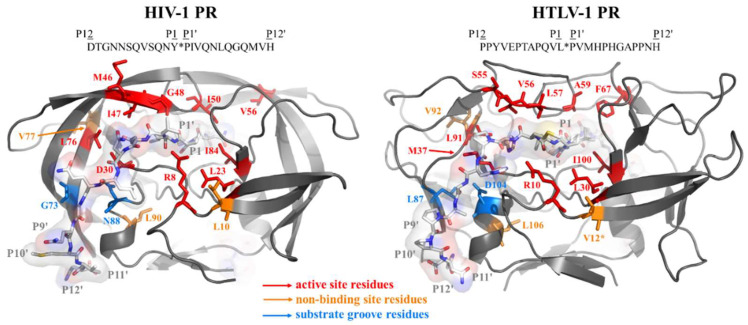
Structures of HIV-1 and HTLV-1 proteases. The modeled structures of HIV-1 and HTLV-1 proteases complexed with substrates representing P12-P12′ residues are shown based on the structural coordinates that were kindly provided by Gary S. Laco [43]. The sequences of the substrates are indicated above the structures. The residues which were modified in this study are shown by sticks, the active site residues which constitute S4-S4’ binding pockets are red, the substrate-groove residues are red, while the residues that are not involved in ligand binding are shown by orange color. * The 12th residue of HTLV-1 PR is valine in the represented structure, but the enzyme we studied in this work contains leucine in this position.

The sequences of the studied proteins were aligned (Figure 1), the mutant BLV and HTLV PRs represented the wild-type or modified residues of HIV-1 PR in the structurally equivalent positions (Figure 2). There is a high structural similarity between the retroviral proteases, although, they exhibit low sequence identity [46]. In agreement with this, the sequence alignment revealed that the BLV and HTLV PRs show only 21–27% sequence identity with HIV-1 PR. The sequence identity shared by HTLV 1/2/3 PRs was higher and ranged from 49–56%, while these proteases showed lower sequence identity with BLV PR (29–33%). The most conserved sequence motifs were found to be the consensus active site motif (D-S/T-G-A) and the consensus short α-helix near the C-terminus (G-R-D, and G-R-N in HIV-1 PR) (Figure 1). Of the studied residues that are in the structurally equivalent positions of the HIV-1, BLV and HTLV PRs (Figure 2) only L30, L76, and L90 are identical in the studied enzymes (according to HIV-1 PR numbering). The L58, A60, and D105 residues are identical in BLV and HTLV PRs (according to BLV PR numbering), but the HIV-1 PR contains different residues in the equivalent positions, while the other residues investigated in this study are less conserved in the corresponding positions among the different enzymes.

Besides the wild-type PRs, enzymes containing stabilizing mutations were also studied. The stabilized form of HIV-1 PR (HIV-1 PR5) contained five mutations: the Q7K, L33I, and L63I mutations in order to minimize the autoproteolysis, and the C67A and C95A mutations to restrict aggregation by preventing cysteine-thiol oxidation [47]. Mahalingam et al., found that the activity of HIV-1 PR5 is indistinguishable from that of the wild-type HIV-1 PR [47]. The stabilized HTLV-1 PR (HTLV-1 PR3) contained three mutations (L40I, C90A, and C109A), the mutations of the Cys residues were designed previously to prevent formation of intermolecular disulphide bonds and the L40I mutation to inhibit autolysis [37]. The kinetic parameters of the wild-type and stabilized enzymes were nearly identical in in vitro activity assays.

A wild-type BLV PR containing mutations of the flanking sequence was also designed (BLV PR2). This recombinant precursor protein contained two mutations of the autoproteolytic cleavage site sequence, both the P2-Cys and P1-Leu residues were changed to Gly. This modification was expected to block self-processing, as BLV PR exhibited considerably lower k_cat_/K_M_ on the peptides containing Gly either in P2 or P1 position as compared to the wild-type [33]. The MBP-fused BLV PR and PR2 differed only in the sequence of the autolytic cleavage site; therefore, the investigation of these enzyme forms was expected to reveal the effect of the N-terminal fusion tag on the self-proteolysis and kinetic characteristics of BLV PR.

### 3.2. Studies on Autoproteolysis

**Figure 3:**
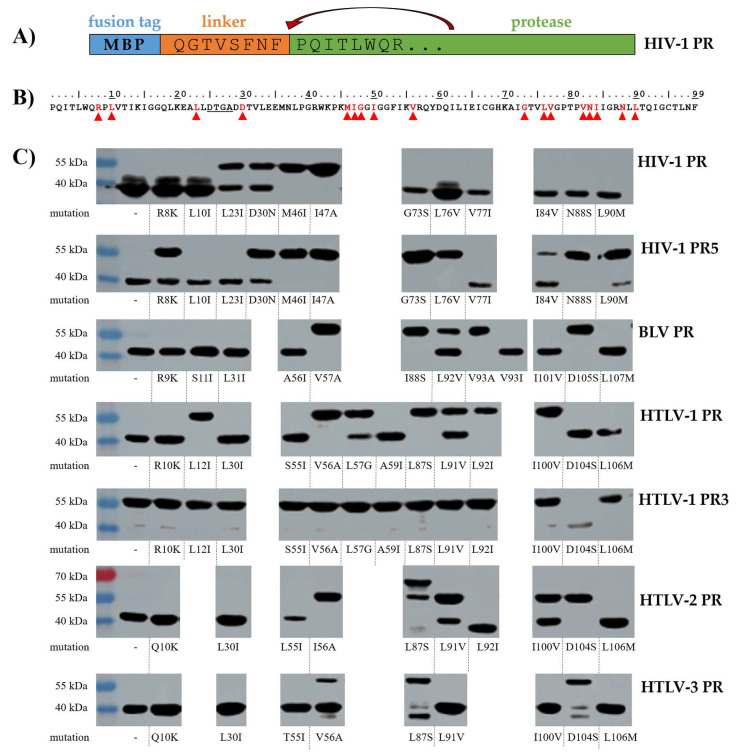
Autoproteolysis of MBP-fused proteases. The proteases were expressed as MBP-fusion proteins, the short linker between the tag and the enzyme represented P8-P1 residues of the natural N-terminal autoproteolytic cleavage site of the protease as exemplified by the wild-type HIV-1 PR (**A**). The effects of protease mutations on the self-processing capability are summarized in Table 2, the studied positions are red and are shown in the sequence of HIV-1 PR by arrowheads (**B**). Representative Western blots are shown based on at least three independent experiments. Anti-MBP antibody was used for Western blot. The bands at higher and lower molecular weights correspond to the full-length MBP-fused proteins and the MBP released via self-processing, respectively (**C**).

The protease precursors were expressed in BL21 (DE3) *E. coli* cells as fusion proteins containing an N-terminal MBP tag. Each construct contained an 8-residue-long sequence at the C-terminus of the MBP that represented the P8-P1 residues of a natural or modified autoproteolytic cleavage site sequence of the given protease. The linker enabled the proteases to process themselves from the fusion forms. The self-processing of wild-type and mutant proteases was followed by detecting the non-processed MBP-fused precursors and the free MBP (released as cleavage product) using anti-MBP antibody for Western blot (Figure 3). 

The efficacy of self-processing was classified based on the degree of protein precursor conversion: (i) the processing was complete if only the MBP (as cleavage product) was detected by Western blot using anti-MBP antibody; (ii) the conversion was considered to be partial if both the full-length fusion protein and the MBP appeared in the blot image; and (iii) the lack of auto-proteolytic activity was indicated if only the MBP-fused protease was detectable on the membrane. For comparison, the set of the single mutations we studied was completed with those ones which have been investigated previously using the same experimental approach, the effects of mutations were also differentiated based on the degree of precursor conversion (Table 2).

#### 3.2.1. HIV-1 PR

Based on the results, the wild-type HIV-1 PR tolerates the mutations well, only the M46I and I47A substitutions—being known major resistance mutations—blocked the self-proteolysis of the MBP-fused protease. The substantial decrease of cleavage efficiency can be explained by the conformational changes of flaps [48] and altered flap opening [49] induced by the M46I mutation. This is in agreement with the detrimental effects of this point mutation which may be compensated by other mutations, although, the virus carrying M46I and other mutations (G48V/L90M) was found to exhibit reduced viral infectivity [50]. We found that the I47A single mutation also prevented the self-proteolysis. This mutation is known to be associated with lopinavir resistance of HIV-1 PR due to the tighter packing of flaps [51] and causes dramatical decrease of the catalytic efficiency (from 100 to 12%) in vitro [52]. In this experimental system we are unable to detect whether this mutant retained any activity, possibly due to its relatively lower sensitivity. Using optimal buffer conditions in vitro, other experimental approaches may be sensitive enough for the detection of residual self-processing activity. Both L23I and D30N mutants showed only partial processing. We expected lower activity of D30N mutant, as it was found to show two-fold decrease of its catalytic efficiency as compared to the wild-type [53]. The activities of the N88S and L90M mutants were comparable and similarly to the wild-type also showed complete turnover, in agreement with the highly similar proteolytic activities of N88D and L90M mutants determined by Kozísek et al. [53]. The HIV-1 PR showed the highest tolerance of the studied enzyme, only 2 of the 12 mutants (M46I and I47A) were incapable of self-processing.

#### 3.2.2. HIV-1 PR5

The same mutations were investigated in the case of the wild-type and the stabilized enzymes (HIV-1 PR and PR5, respectively). In the absence of additional mutations both HIV-1 PR and PR5 were capable for complete self-processing; however, an additional band over the processed HIV-1 PR suggested the presence of an inactive, somewhat extended MBP-truncated protease form, presumably due to the degradative cleavage at L33. This is not observed in the PR5 cleavage pattern. L10I and V77I mutants as well as D30N retained their full or partial autoprocessing capability, respectively. While the self-processing was complete for the R8K, I84V, and L90M mutant HIV-1 PRs, it was only partial for the same mutants of HIV-1 PR5. This is in agreement with the previous observations, the R8Q and L90M mutations were found to decrease the catalytic efficiency of HIV-1 PR5 on a substrate representing p6^pol^/PR cleavage site [47]. The most substantial decrease of activity was observed for the G73S, L76V and N88S mutations which prevented the self-processing of HIV-1 PR5, although, the HIV-1 PRs containing the same mutations retained their activity and showed complete turnover. The only mutant showing higher self-processing capability in the stabilizing mutation background was L23I. Overall, the mutation tolerance of HIV-1 PR5 was lower as compared to that of the wild-type. It should be noted that while the N88S mutant HIV-1 PR5 did not exhibit self-proteolysis in this study (Figure 3), the N88D mutant was found to retain its activity with decreased catalytic efficiency (the k_cat_/K_M_ was 20–40% of the wild-type, depending on the applied substrate) [47]. Therefore, in vitro kinetic assays may be sensitive enough to detect the residual activities of G73S, L76V, and N88S mutants which showed no activity in this self-processing assay.

#### 3.2.3. BLV PR

In total, 44% of the previously or herein investigated mutations of BLV PR were neutral and did not alter self-processing as compared to the wild-type (R9K, S11I, L31I, A56I, V93I, W99V, I101V, and L107M). The N38D, V57I, and L92V mutations decreased cleavage efficacy, while 7 of the 18 studied mutations (V57A, L58G, A60I, I88S, V93A, K98P, and D105S) prevented the processing (Table 2). The substitutions of the wild-type BLV PR residues to those of the wild-type HIV-1 PR caused decreased (N38D, V57I) or abolished activity (L58G, A60I, K98P), with the exception of W99V mutation which exhibited no change. Interestingly, the effects of mutations at 57th and 93rd positions were different. The introduction of an Ile residue into BLV PR (V57I and V93I) did not prevent self-processing but showed only partial rather than complete turnover. In contrast to this, the V57A and V93A mutants did not undergo self-proteolysis. Like the I47A mutation of HIV-1 PR and PR5, the Ala-substitution in the equivalent position (V57A) of BLV PR prevented self-processing. The V93I mutant BLV PR showed no difference as compared to the wild-type, the turnover was complete, similar to the modified HIV-1 PR that contained Ile in the same position (V77I). Interestingly, the A56I mutation of the flap did not alter the self-processing ability, although, both HIV-1 PR and PR5 were unable for auto-proteolysis (Table 2), while the mutation in the neighboring position (I47A according to HIV-1 PR numbering) abolished ability for self-processing in all cases, excepting HTLV-3 PR. In total, 39% of the mutants were defective for self-proteolysis, which implies lower mutation tolerance of BLV PR as compared to the wild-type HIV-1 PR.

#### 3.2.4. HTLV-1 PR

The mutations of L12, M37, V56, F67, L87, V92, and I100 residues blocked the self-processing of the wild-type HTLV-1 PR, while most of the other substitutions were neutral, latter mutants exhibited complete turnover (Table 2). The A59I mutant HTLV-1 PR showed complete processing, although, our former kinetic study implied that it is a very inefficient enzyme as compared to the wild-type [34]. The L57G mutant also retained its activity but showed only partial turnover. Interestingly, this mutant was found previously to unable to hydrolyze the oligopeptide representing CA/NC cleavage site (KTKVL↓VVQPK) [34]. This difference may be caused by the strong dependence on the sequence context of the substrate, the best substrate of the L57G mutant was the P3 Ala-substituted peptide while the non-modified CA/NC peptide was not hydrolyzed [34]. The V56A mutant was one of the non-processing mutants. Accordingly, kinetic analyses revealed that the V56I mutant is also a very inefficient enzyme [34]. The Ala-substitution in the structurally equivalent position prevented processing of all investigated enzymes, excepting HTLV-3 PR (Table 2). Residue V56 (according to HTLV-1 PR numbering) is part of the flap, it is known to interact with P2 and P4 residues of the substrate [45]. In the studied HTLV PRs, the self-processing sites are highly similar and show the most remarkable differences at P4 position (Figure 1), but without extended structural analysis it can hardly be estimated whether the differences of the cleavage site sequences or the flap regions cause only HTLV-3 PR to retain its activity upon the mutation at 56th position. The ability of S55I mutant for self-processing resembled that of the wild-type HTLV-1 PR, and similarly to respective A56I, L55I and T55I mutant BLV, HTLV-1 and HTLV-2 PRs showed complete turnover. Of the wild-type enzymes, the Ile-substitution in the structurally equivalent position (M46I) abolished the self-processing ability only in the case of HIV-1 PR (Table 2). In total, >50% of the HTLV-1 PR mutants were found to be non-processing; thus, the HTLV-1 PR showed the lowest mutation tolerance of the wild-type enzymes. 

#### 3.2.5. HTLV-1 PR3

The HTLV-1 PR containing stabilizing mutations (HTLV-1 PR3) appeared to have defective self-processing ability, indicating improper folding and/or activity of the folded mutants. We detected autoproteolytic activity only for the D104S mutant enzyme (complete turnover). The D104S mutant HTLV-1 PR3 contains the mutation of the S-groove, we assume that the mutant residue may contribute to the enzyme-substrate interactions at the enzyme surface in such extent that makes the enzyme capable for self-hydrolysis. The effect of D104S mutation of HTLV-1 PR3 may be similar to that of the N88D mutation of HIV-1 PR, which mutant residue was found to form stronger interactions with the substrate, increasing the interactions with the P7 and P7′ residues [43], but without extended structural analyses of the effects of the D104S mutation in HTLV-1 PR3 can be hardly estimated. In our experimental system only HTLV-1 PR showed activity, the HTLV-1 PR3 was not capable for self-proteolysis (Figure 3) (Table 2). The lack of auto-proteolysis may be caused by the suboptimal conditions for enzymatic activity in the bacterial cells, because the activity and specificity of HTLV-1 PR3 was found to be identical with those of the wild-type HTLV-1 PR, using more optimal buffer conditions in vitro [37]. It should also be mentioned, that the HTLV-1 PR3 utilized for kinetic and inhibition profiling was expressed not as a fusion protein but rather as the mature enzyme form that was untagged, and it was successfully refolded after purification from inclusion bodies [37]. It should be noted that the TF1/PR autoproteolytic cleavage sites of the MBP-fused proteins were also slightly different, the HTLV-1 PR3 contained Ala residue in P4 position, while this residue was Thr in the case of HTLV-1 PR. This difference may also somewhat interfere with the altered cleavage efficacy, as the mutation of Thr to Ala in P4 position caused ~4.5-fold decrease of the HTLV-1 PR3′s catalytic efficiency, in the context of HTLV-1 CA/NC cleavage site [54]. Both HTLV-1 PR and PR3 contained an N-terminal flanking sequence prior to the MBP tag representing the HTLV-1 TF1/PR cleavage site. This cleavage site (DPASIL↓PVIP) has already been used to study the specificity of HTLV-1 PR and PR3 [34,35,37], and the lowest cleavage efficiency was obtained for this TF1/PR substrate as compared to MA/CA, CA/NC and PR/p3 cleavage sites. The catalytic constant was significantly lower in case of TF1/PR, the obtained k_cat_/K_M_ values were at least an order of magnitude lower (25–76 and 22–75-fold lower for HTLV-1 PR and PR3, respectively) [37].

#### 3.2.6. HTLV-2 PR

Similar to HTLV-1 PR, the L37D, L37N, I56A, F67Q, and L87S mutations prevented self-processing of HTLV-2 PR, as well (Table 2). We observed partial turnover only for L91V and I100V mutants. In contrast to HTLV-1 PR, the I100V mutation did not inactivate HTLV-2 PR. The L55I mutant showed no altered activity as compared to the wild-type, while the HIV-1 PR containing mutation in the corresponding position of the flap (M46I) showed no self-processing. In the case of L87S mutant HTLV-2 PR, we detected two bands, one at the expected molecular weight of the MBP-fused HTLV-2 PR and another one at higher molecular weight. While no detectable band appeared at the molecular weight of the self-processed protease, the processing of this mutant cannot be determined unequivocally; therefore, we assume that this enzyme did not undergo self-proteolysis. The overall tolerance of HTLV-2 PR was similar to that of the HTLV-1 PR, 40% of the mutants showed no activity.

#### 3.2.7. HTLV-3 PR

Only the previously studied mutations were found to prevent self-processing of the enzyme, the mutants which were designed in this work retained their activity (Table 2). The F67Q and I37D mutations prevented processing of all HTLV PRs, and the HTLV-3 PR was the only which tolerated the I37N mutation. Based on the appearance of the proteolytic cleavage products at lower-than-expected molecular weights, we assumed the possible shift of the cleavage position in the case of L92I mutant HTLV-2 PR (at complete auto-proteolysis) and for L87S and D104S mutant HTLV-3 PRs (at partial self-processing) (Figure 3). A similar shift of the cleavage position was detected previously for the N38D mutant BLV PR [33]. The double product band in the case of D104S mutant HTLV-3 PRs implies that cleavage may occur not only at a single but at two sites. As compared to BLV and HTLV-1/2 PRs, the number of the mutations which inhibited auto-proteolysis of HTLV-3 PR was considerably lower.

### 3.3. Autoproteolysis, Kinetic Analysis and Inhibition of BLV PR2

**Figure 4:**
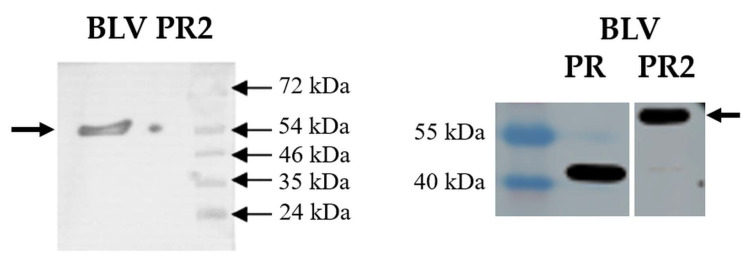
Autoproteolysis of BLV PR2. The Western blot was performed by using anti-BLV (left panel) and anti-MBP antibody (right panel). Arrows indicate the full-length MBP-fused proteins, self-proteolysis was detected only in case of the wild-type enzyme.

The MBP-fused BLV PR containing Gly residues in the P2 and P1 positions of the cleavage site (BLV PR2) was unable for self-processing (Figure 4), as it was expected based on the previously established specificity of the BLV PR [33].

Although BLV PR2 was unable for self-processing, we conducted the investigation of the activity of this cleavage site mutant MBP-fused precursor and the comparison of the kinetic parameters to that of the untagged enzyme (BLV PR). The kinetic analysis was expected to reveal the effect of the N-terminal MBP fusion tag on the enzymatic activity. For kinetic analyses, we applied an oligopeptide-based in vitro activity assay, which was considered to be more sensitive due to the buffer conditions that are more optimal for the enzymatic activity as compared to the cellular environment of the bacterial cells. 

Both BLV PR and PR2 were active in the in vitro activity assay and had 100% folding efficiency based on the ratio of the active enzyme and the total protein amount (Table 3). This proved that the simultaneous Gly-substitutions at P2 and P1 positions prevent self-processing but did not interfere with the protein folding. Accordingly, the BLV PR2 was subjected for kinetic analyses. 

The kinetic parameters were determined using an oligopeptide substrate representing HTLV-1 CA/NC cleavage site (Table 3). The K_M_ values were almost identical for BLV PR and PR2. The catalytic constant (k_cat_/K_M_) obtained for the wild-type was comparable with those determined previously for HIV-1 and HTLV PRs, while the k_cat_/K_M_ of BLV PR2 was 6-fold lower as compared to the wild-type BLV PR. Our results proved that the presence of the N-terminal MBP interferes with the activity and decreases the catalytic efficiency of the BLV PR. Despite having lower activity as compared to the untagged wild-type, the MBP-fused BLV PR2 may be used in activity assays, such as inhibition studies. To prove this, we determined the inhibitory potential of IB-268 inhibitor against BLV PR2 and compared it to the inhibitory constant that was determined previously for the untagged BLV PR.

The IB-268 was found previously to be a very weak inhibitor of HIV-1 PR and showed inhibition only in micromolar concentration range (K_i_: >11 µM) [55], but it inhibited the BLV [33] and HTLV PRs [35,55] more efficiently. Based on the K_i_ values determined previously for the BLV, HIV-1, and HTLV PRs, the IB-268 was the most potent inhibitor of BLV PR, but interestingly, we obtained an unusually low K_i_ value for BLV PR2 which was 3.3-fold lower than we determined for the wild-type (Table 3).

## 4. Discussion

In this work we studied the autoprocessing of the MBP-fused precursor forms of BLV, HTLV-1, HTLV-2, and HTLV-3 deltaretroviral PRs and the abilities of the wild-type and modified enzymes were compared to those of lentivirus HIV-1 PRs. The PRs were expressed fused to an N-terminal MBP, the precursor proteins contained an N-terminal flanking sequence prior to the protease domain which linker represented autoproteolytic cleavage site sequences. Natural sequence variants or single drug resistance mutation-containing HIV-1 PR mutants were prepared, and we designed such mutant BLV and HTLV PRs which represented the wild-type or modified residues of HIV-1 PR in the structurally equivalent positions. HIV-1 and HTLV-1 PRs containing stabilizing mutations were also studied, as these enzyme forms are generally used to investigate resistance mutations in vitro. 

The autoproteolysis assays—using the MBP-fused precursors—revealed different mutation tolerance of wild-type BLV and HTLV deltaretroviral PRs as compared to HIV-1 PR. The highest mutation tolerance was observed for HIV-1 PR, only <15% of the mutations prevented self-proteolysis. The wild-type deltaretroviral proteases (BLV, HTLV-1, HTLV-2, and HTLV-3) showed higher sensitivity as compared to the HIV-1 PR, the percentage of the non-processing mutants was the lowest for HTLV-3 PR (21.4%). The observed differences between the abilities of HIV-1 and deltaretroviral PR precursors for self-processing correlate with the distinct replication strategies of these viruses.

Enzymes containing stabilizing mutations, such as the HIV-1 PR5 [56,57,58,59,60] and HTLV-1 PR3 [34,35,39,61,62] are used in in vitro assays; therefore, we investigated precursors of stabilized proteases, as well. The stabilized enzymes showed considerably lower mutations tolerance, the number of non-processing mutants was higher in case of the HIV-1 PR5 containing stabilizing mutations as compared to the wild-type (41.7% and 14.3%, respectively), while the wild-type HTLV-1 PR3 was defective for self-processing in almost all cases. 

The mutations at the active site, in the S-groove or in the hydrophobic core (e.g., L90 residue of HIV-1 PR) may alter folding efficiency; consequently, many mutants may exhibit decreased activity [33,34]. But, modification of the N-terminal flanking sequence can influence self-processing without altering folding of the enzyme itself, as we have seen this in case of BLV PR2 where the simultaneous Gly-substitutions at P2 and P1 positions of the self-cleavage site prevented the processing. The folding efficiency of the mutant and the wild-type was 100%, the catalytic constant obtained for the mutant was lower, due to the presence of the MBP at the N-terminus of the protease which decreased the activity as compared to the untagged enzyme. Despite showing no autoproteolytic activity, the BLV PR2 was efficiently inhibited by IB-268 inhibitor, the obtained K_i_ value was remarkably lower as compared to the wild-type BLV PR.

In summary, our results support the hypothesis that the BLV and HTLV deltaretroviral PRs did not undergo such extensive mutational changes that might have optimized the HIV-1 PR for high catalytic efficiency. In addition, the HIV-1 PR has a higher flexibility in tolerating mutations under selective pressure. Despite the relatively lower sensitivity of the herein applied experimental system and the different catalytic properties of the non-purified and in vitro-refolded enzymes, the recombinant precursors can be used efficiently to differentiate the mutations in larger scale, followed by investigation of catalytic properties using more sensitive in vitro activity assays. Our results shed light on the limitations of the self-proteolysis assays that are based on the use of MBP-fused precursors containing stabilizing mutations, which needs to be considered while studying drug-resistance mutants and screening PR inhibitors using this experimental approach. 

## Figures and Tables

**Table 1:** Expression constructs. The recombinant proteins expressed from the plasmids contained an N-terminal MBP fusion tag, the protease was connected to this tag via an 8 residue-long linker representing the natural cleavage site of the given protease. The arrows indicate cleavage position within the autoproteolytic cleavage site sequences. Cleavage site mutations are bold and underlined.

Protease	Plasmid	Cloning Site	Protease Mutation	N-terminal Auto- Proteolytic Sequence	Reference
BLV PR	pMalc2x	EcoRI/SalI	no	SEAELECL↓LSIPLARS	[38]
BLV PR2	pMalc2	EcoRI/SalI	no	SEAELE**GG**↓LSIPLARS	this study
HIV-1 PR	pMalc5x	NdeI/BamHI	no	QGTVSFNF↓PQITLWQR	[36]
HIV-1 PR5	pMalc5x	NdeI/BamHI	Q7K, L33I, L63I, C67A, C95A	QGTVSFNF↓PQITLWQR	this study
HTLV-1 PR	pMalc2x	EcoRI/BamHI	no	NQDPTSIL↓ PVIPLDPA	[34]
HTLV_1 PR3	pMalc5x	NdeI/BamHI	L40I, C90A, C109A	NQDP**A**SIL↓ PVIPLDPA	this study
HTLV-2	pMalc2x	EcoRI/BamHI	no	PDQDISIL↓ PLIPLRQQ	[35]
HTLV-3	pMalc2x	EcoRI/BamHI	no	LTSPRTIL↓PLIPLSQQ	[35]

**Table 2:** Capabilities of the MBP-fused proteases for autoproteolysis. The effects of single point mutations are shown based on the results of Western blot experiments (Figure 3). The herein studied set of mutants was completed with those that were studied previously: ^1^ [33], ^2^ [34], and ^3^ [35]. Asterisk indicates that a previously studied mutation was investigated in this work as well. Complete and partial self-processing is indicated by green and yellow colors, respectively, while red background indicates that the mutation blocked autoproteolysis. n.d.: the effect of the corresponding mutation in the given position was not determined (grey background).

HIV-1 PR	HIV-1 PR5	BLV PR	HTLV-1 PR	HTLV-1 PR3	HTLV-2 PR	HTLV-3 PR	Modified Position
wt	wt	wt ^1,^*	wt ^2,^*	wt	wt ^3,^*	wt ^3,^*	-
R8K	R8K	R9K	R10K	R10K	Q10K	Q10K	S3, S1
L10I	L10I	S11I	L12I	L12I	I12 (wt) ^3,^*	I12 (wt) ^3,^*	-
L23I	L23I	L31I	L30I	L30I	L30I	L30I	S1
D30 (wt)	D30 (wt)	N38D ^1^	M37D ^2^	n.d.	L37D ^3^	I37D ^3^	S4, S2
D30N	D30N	N38 (wt) ^1,^*	M37N ^2^	n.d.	L37N ^3^	I37N ^3^	
n.d.	n.d.	n.d.	M37A ^2^	n.d.	n.d.	n.d.	
M46I	M46I	A56I	S55I	S55I	L55I	T55I	S4
I47A	I47A	V57A	V56A	V56A	I56A	V56A	S4, S2
I47 (wt)	I47 (wt)	V57I ^1^	n.d.	n.d.	n.d.	n.d.	
G48 (wt)	G48 (wt)	L58G ^1^	L57G	L57G	L57G ^3^	L57G ^3^	S4, S3, S2
I50 (wt)	I50 (wt)	A60I ^1^	A59I	A59I	A59I ^3^	A59I ^3^	S3, S2, S1
V56 (wt)	V56 (wt)	Y68 (wt) ^1,^*	F67Q ^2^	n.d.	F67Q ^3^	F67Q ^3^	S4
G73S	G73S	I88S	L87S	L87S	L87S	L87S	S-groove
L76V	L76V	L92V	L91V	L91V	L91V	L91V	S4
V77I	V77I	V93I	V92I	V92I	L92I	I92 (wt) ^3,^*	-
n.d.	n.d.	V93A	n.d.	n.d.	n.d.	n.d.	
P82 (wt)	P82 (wt)	K98P ^1^	n.d.	n.d.	n.d.	n.d.	S1
V83 (wt)	V83 (wt)	W99V ^1^	n.d.	n.d.	n.d.	n.d.	S1
I84V	I84V	I101V	I100V	I100V	I100V	I100V	S2, S1
N88S	N88S	D105S	D104S	D104S	D104S	D104S	S-groove
L90M	L90M	L107M	L106M	L106M	L106M	L106M	-
12	12	18	17	13	15	14	Total number of mutants
2/12 14.3%	5/12 41.7%	7/18 38.9%	9/17 52.9%	12/13 92.3%	6/15 40.0%	3/14 21.4%	Number and percentage of non-processing mutants

**Table 3:** Comparison of the catalytic properties and folding efficiencies. The kinetic parameters and folding efficiencies are shown for HIV-1, BLV and HTLV PRs. For kinetic analysis of MBP-fused BLV PR2, we used oligopeptide substrate representing HTLV-1 CA/NC cleavage site. Average values are shown based on the results of two independent experiments, the errors are indicative and represent differences between the obtained values. For comparison, literature data are also shown: the values for the wild-type and N38D mutant BLV PRs ^1^ [33], and for HTLV and HIV-1 PRs were determined previously ^2^ [55] ^3^ [54] ^4^ [37] ^5^ [35]. ^#^ The K_M_ and k_cat_ values were also determined previously by Kassay et al. [35] but have not been published to date.

		K_M_ (mM)	k_cat_ (s^−1^)	k_cat_/K_M_ (mM^−1^s^−1^)	Folding Efficiency	K_i_ (nM)
**BLV**	PR	0.011 ^1^	0.27 ^1^	24.5 ^1^	100% ^1^	13 ^1^
	PR-N38D	0.18 ^1^	0.023 ^1^	0.13 ^1^	21% ^1^	-
	PR2	0.014 ± 0.002	0.058 ± 0.002	4.14 ± 0.61	100%	3.93
**HIV-1**	PR5	0.16 ^3^	3.6 ^3^	22.5 ^3^	100% ^3^	11.215 ^2^
**HTLV-1**	PR3	0.063 ^3^	10.0 ^3^	158.7 ^3^	100% ^3^	298 ^2^
		0.051 ± 0.005 ^4^	7.68±0.17 ^4^	150.6 ^4^	-	-
**HTLV-2**	PR	0.168 ± 0.078 ^#^	11.27 ± 1.932 ^#^	67.2 ^5^	-	37 ^5^
**HTLV-3**	PR	0.144 ± 0.066 ^#^	4.344 ± 0.985 ^#^	30.1 ^5^	-	214 ^5^

## Data Availability

The data presented in this study are available on request from the corresponding author.

## Supplementary Materials

The following supporting information can be downloaded at: https://www.mdpi.com/article/10.3390/v14091888/s1, Table S1: List of oligonucleotide primers used for mutagenesis.
